# Opportunities and challenges to engineer 3D models of tumor-adaptive immune interactions

**DOI:** 10.3389/fimmu.2023.1162905

**Published:** 2023-04-04

**Authors:** Rahul M. Visalakshan, Mary K. Lowrey, Mauricio G. C. Sousa, Haylie R. Helms, Abrar Samiea, Carolyn E. Schutt, Josh M. Moreau, Luiz E. Bertassoni

**Affiliations:** ^1^ Knight Cancer Precision Biofabrication Hub, Knight Cancer Institute, Oregon Health and Science University, Portland, OR, United States; ^2^ Cancer Early Detection Advanced Research Center, Oregon Health and Science University, Portland, OR, United States; ^3^ Division of Biomaterials and Biomechanics, Department of Restorative Dentistry, School of Dentistry, Oregon Health & Science University, Portland, OR, United States; ^4^ Department of Biomedical Engineering, School of Medicine, Oregon Health and Science University, Portland, OR, United States; ^5^ Division of Oncological Sciences, Oregon Health and Science University, Portland, OR, United States; ^6^ Department of Dermatology, Oregon Health and Science University, Portland, OR, United States

**Keywords:** 3D *in vitro* models, cancer adaptive immunity, organoids, bioprinting, organs on a chip

## Abstract

Augmenting adaptive immunity is a critical goal for developing next-generation cancer therapies. T and B cells infiltrating the tumor dramatically influence cancer progression through complex interactions with the local microenvironment. Cancer cells evade and limit these immune responses by hijacking normal immunologic pathways. Current experimental models using conventional primary cells, cell lines, or animals have limitations for studying cancer-immune interactions directly relevant to human biology and clinical translation. Therefore, engineering methods to emulate such interplay at local and systemic levels are crucial to expedite the development of better therapies and diagnostic tools. In this review, we discuss the challenges, recent advances, and future directions toward engineering the tumor-immune microenvironment (TME), including key elements of adaptive immunity. We first offer an overview of the recent research that has advanced our understanding of the role of the adaptive immune system in the tumor microenvironment. Next, we discuss recent developments in 3D *in-vitro* models and engineering approaches that have been used to study the interaction of cancer and stromal cells with B and T lymphocytes. We summarize recent advancement in 3D bioengineering and discuss the need for 3D tumor models that better incorporate elements of the complex interplay of adaptive immunity and the tumor microenvironment. Finally, we provide a perspective on current challenges and future directions for modeling cancer-immune interactions aimed at identifying new biological targets for diagnostics and therapeutics.

## 1 Introduction

For many years, cancer biology was studied on two-dimensional (2D) culture dishes. While this method has yielded extremely useful insights into the behavior of tumor cells, it is well established that 2D models do not accurately reflect the complex tumor–host interactions that occur in patients, or even in animal models (1, 2). As a result of this limitation, many promising preclinical and basic research findings fail to translate into meaningful clinical results (3). Tumor-derived cell lines and mouse models have been used to understand cancer processes and stages for over a century. Tumor-derived cell lines are advantageous because they are easy to maintain in culture, represent important features of certain cancers, and can be useful in unraveling biochemical pathways (4). However, these cells often have different genetic profiles from those of the primary cells derived from patients. Additionally, cross contamination with other lines is possible as well as genetic drift occurring in long term cultures (5). Most tumors are heterogeneous, and the process of direct and indirect interactions between the cell-cell and the cell environment is of paramount importance in their development, invasion, and metastasis (6). However, existing experimental models with conventional primary cells or cell lines have been found to be inadequate for studying these complex systems (7).

Mouse models that mimic human cancers have also increased over the last few decades; however, one major limitation with using mouse models for studying cancer immunology is the difficulty in accurately replicating the human immune system and tumor microenvironments due to differences between species anatomy and physiology (8). This can lead to results that are not applicable when attempting clinical translation, since they may lack relevance due to being unable to replicate certain features present in humans that are absent from mice (9, 10). This may include specific receptors/ligands expressed only by human cells or epigenetic modifications caused by environmental exposure only found among humans (11), which cannot be replicated through animal testing methods (4, 12). In addition, fast growth of syngeneic tumors and the need for mouse-adapted surrogate antibodies or biologicals limit the usefulness of these models. Excessive fast growth of typical syngeneic tumors in mouse models can result in rapid progression and metastasis, leading to difficulties in evaluating the efficacy of potential therapies. Furthermore, the use of mouse-adapted surrogate antibodies or biologicals may not accurately reflect the interactions between human cells and immunotherapeutic agents and further complicate the translation of findings to human patients (13–15).

Recent advances in tissue engineering have enabled researchers to create 3D structures that more closely resemble native tissues than traditional 2D cultures. This advancement allows for improved control over environmental factors that can influence cancer progression. In addition, engineered microenvironments enable researchers to study cellular behaviors at different scales, from individual cells, all the way up through organoids/tumoroids (16). These technologies also provide an opportunity for creating more physiologically relevant models by incorporating patient specific data into exogenously controlled environments made from biomaterials such as hydrogels or scaffolds seeded with various types of cancerous cells (17).

3D *in vitro* models may provide a viable solution as they can help reduce animal experiments during preclinical studies, accelerate therapeutic target discovery, cut down R&D spending and potentially decrease spending on global cancer care (18, 19). By creating functional tissue structures that accurately mimic human biology, 3D *in vitro* models allow researchers not only to study disease progression, but also test different therapeutics directly *in vitro*, which could drastically shorten drug development timelines (20). Furthermore, since these models are more accurate than traditional cell cultures, they enable personalized medicine allowing clinicians to better predict patient response to specific treatment protocols thus reducing healthcare expenditure while improving overall outcomes.

The use of 3D cell cultures can help recreate complex architectures, such as tumor microenvironments, by incorporating both innate and adaptive immunity components, along with other biological events that occur during tumor progression, or even metastasis (21, 22). Furthermore, these tools can also be used in combination with gene editing technologies, like CRISPR/Cas9 or RNA interference (RNAi) screening methods, which allow researchers to identify pathways involved in immunotherapy response or resistance when testing novel therapies against specific diseases such as lung cancer or breast cancer cells at various stages throughout their development process (23, 24).

In recent years there has been an increased focus on understanding how three-dimensional (3D) microenvironments can better replicate the complexities seen within living systems. By incorporating elements like perfusable vasculature, 3D tumor architecture, multiple stromal cell types and immune components found within a natural TME environment, researchers are able to gain more accurate insights about how different treatments may affect cancer progression over time (17). These methods also make it possible to determine which therapies are most likely to be successful for individual patients based on their unique tumor profiles. Recent studies suggest that *ex vivo* 3D printed tumor models may already replace existing two-dimensional cell cultures on rigid plastic plates, as they better replicate complex structures composed of different cell types found within solid tumors, such as breast or prostate cancers (18). Furthermore, they can be used to personalize anticancer therapies based on individual patient characteristics by generating personalized tissue constructs from primary cells obtained directly from patients using biopsy samples (17, 25). This could potentially reduce reliance on animal testing. Overall novel 3D models offer a promising solution towards understanding tumor heterogeneity on a deeper level than was previously possible using conventional techniques alone (26).

Despite these collective promises, there are still significant challenges towards broad adoption of 3D engineered constructs as a preferred tool in cancer immunity research. In this review, we discuss the current state of research on 3D modeling approaches and their potential implications for advancing our understanding of tumor immunology and developing effective cancer treatments and 3D models of tumor-adaptive immune interactions.

The equipment and expertise required for 3D *in vitro* models can vary depending on the specific model being used. Some models, such as hanging drop and spheroid cultures, require relatively simple equipment such as cell culture plates, pipettes, and centrifuges. On the other hand, more complex models such as organoids and bioprinted constructs may require specialized equipment such as bioreactors, microfluidic devices, and 3D bioprinters (27). Despite their potential advantages, 3D *in vitro* models also have several limitations and challenges that may affect their broader applicability. First and foremost, traditional cancer biology and immunology labs may not have the engineering experience and skills that are required to design and develop these model systems (28). Nevertheless, the popularization and expanding commercialization of ‘off-the-shelf’ micro-physiological microdevices, bioprinters, and organoid engineering tools, is rapidly changing the landscape at the interface of engineering and biology (29). Moreover, the architecture and cellular interactions that occur *in vivo* are not always completely replicated in 3D models, and this is a challenge that needs to be addressed by the engineering team generating such models. Additionally, culturing and maintaining some 3D models can be difficult and may require specialized media and culture conditions. The lack of standardization among 3D models and the variations in their preparation methods also pose challenges in terms of reproducibility and comparability of results (25). Finally, the cost of some of the specialized equipment and materials required for 3D *in vitro* models may limit their broader use and accessibility, particularly in resource-limited settings. Despite these challenges, researchers have made significant strides in developing and optimizing 3D models to better mimic human tumors and their microenvironment, such as the use of patient-derived organoids or microfluidic systems that enable perfusion and dynamic culture conditions (30, 31). With continued advances in technology and methods, 3D *in vitro* models have the potential to become an important tool for preclinical drug screening and personalized medicine.

## 2 Key elements of adaptive immunity in solid tumors

### 2.1 Adaptive immunity in solid tumors

**Figure 1:**
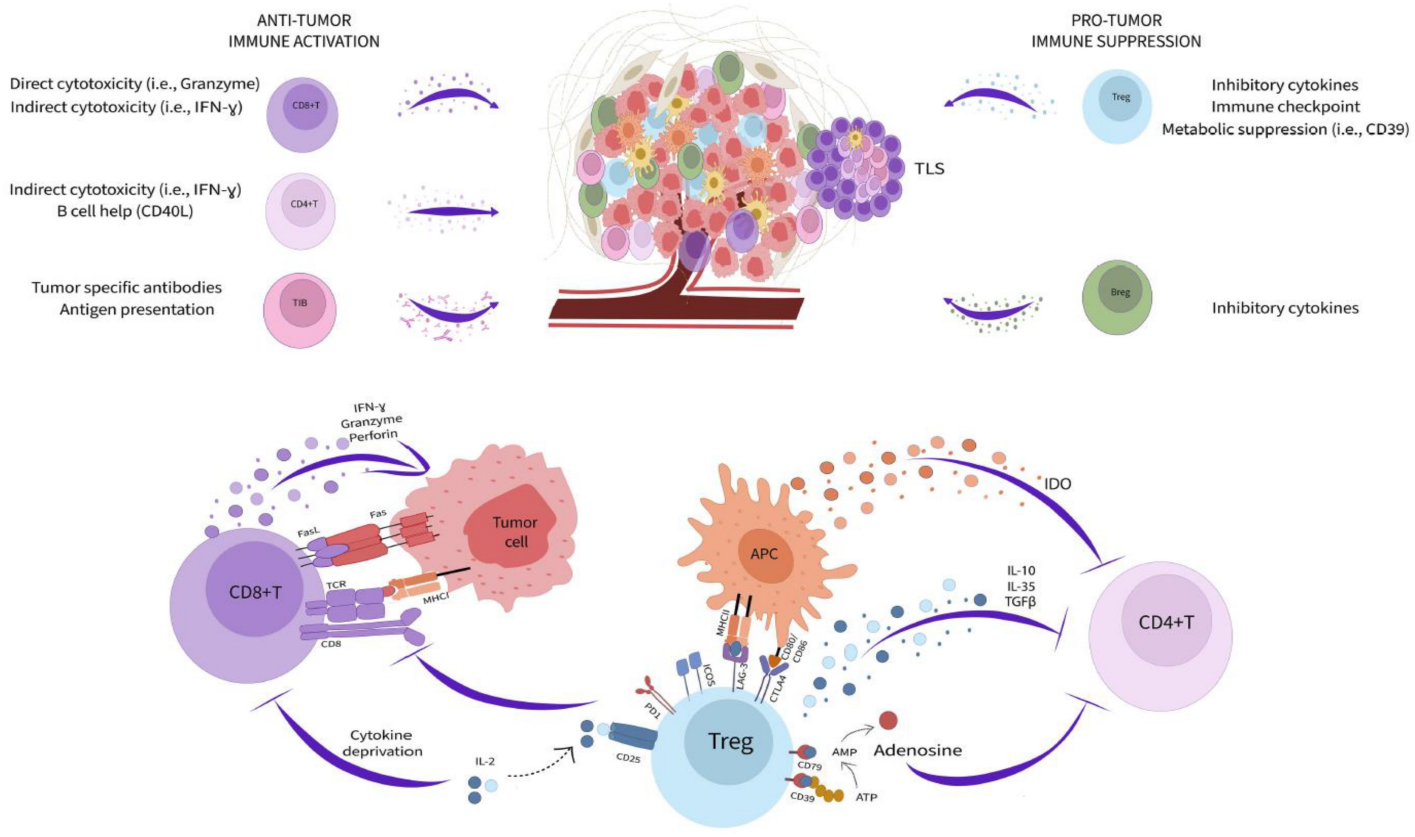
Adaptive immunity in the tumor microenvironment. Subsets of adaptive immune cells accumulating within the TME kill cancer cells through a variety of cytotoxicity mechanisms (left side) while other populations (right side) impair immune function and suppress cytotoxic activity contributing to continued tumor expansion. Tregs mediate immunosuppression within the TME through multiple pathways (bottom).

Adaptive immunity is inextricably linked to cancer progression. The impressive clinical success of immune checkpoint blockade (ICB) capitalizes on fundamental mechanisms of T cell regulation and highlights the opportunity in modulating adaptive immune biology for cancer therapy. However, many patients and certain tumor types are poor responders to these treatments (32). Moreover, the activity of T and B cells within the tumor microenvironment (TME) is enormously complex and still incompletely understood. Immune function in cancer is highly regulated by local cellular interactions (**Figure 1**). Several subsets of adaptive immune cells accumulate within the TME. In some tumors, stromal cells modulate extracellular matrix composition to impede B and T cell infiltration leading to poor patient prognosis and reduced response to immunotherapy (33–35). While immune accumulation is an important attribute of anti-cancer immunity, it is insufficient for protection. Cancer cells, fibroblasts, and regulatory immune populations impair immune function and suppress cytotoxic activity contributing to continued tumor expansion.

Among the most potent immunoregulatory cells are regulatory T cells (Treg) and B cells (Breg). Tregs are a discrete CD4^+^ T cell lineage whose accumulation is closely correlated with negative clinical outcomes (36, 37). They utilize an extensive toolkit of molecular mediators to modify the TME and attenuate anti-cancer immunity. Tregs, both secrete and activate latent TGF-β, produce additional inhibitory cytokines, including IL-10 and IL-35, and due to their high expression of CD25, reduce the local availability of IL-2 (36, 37). They also modulate TME metabolic activity through expression of CD39 and CD73, that convert ATP into adenosine, which both suppresses immune cells and promote a pathogenic activity among tumor associated fibroblasts (38, 39). Tregs additionally maintain suppression through immune checkpoint pathways, such as PD-1/PD-L1, ICOS/ICOSL, LAG-3/MHC class II, and CTLA-4 (36, 37). These molecules disrupt antigen presentation, T cell activation, and T cell co-stimulation ultimately promoting a dysfunctional and anergic cell state that profoundly neutralizes effector T cell activity within the TME (32). In contrast to Tregs, Bregs are not a defined cell subset, and instead reflect a B lymphocyte cell state driven by as yet unclear local environmental cues (40). Nonetheless, Bregs mediate immunosuppression through similar pathways to Tregs, especially production of the cytokines IL-10, IL-35, and TGF-β (40). Small populations of CD8^+^ regulatory T cells have also been reported but the contribution these cells to the TME is unclear (41, 42). Strategies to reduce the potency of regulatory cells is an important goal for cancer therapy and has already yielded positive results. For example, CTLA-4 blockade, which has clinical efficacy in several cancers, works in large part through direct Treg depletion (32, 43).

Despite the many pathways dampening immunity within solid tumors, T and B cells retain enormous anti-cancer capacity. The accumulation of both lineages in the TME correlates with positive prognosis across cancer types and the abundance of CD8^+^ T cells predicts ICB response in metastatic melanoma (44–46). Patients successfully responding to ICB frequently exhibit rapid cancer cell death and durable disease clearance (32). CD8^+^ T cells are key targets of ICB and powerful mediators of cancer killing. These cells are loaded with direct cytotoxic mediators, including granzymes, perforin, interferon gamma (IFNγ), and Fas Ligand, that allow them to serially kill cancer cells displaying cognate antigens within only few minutes of interaction (47–50). Effector CD4^+^ accumulating in the TME also support cancer immunity through the secretion of inflammatory cytokines (*i.e.*, IFNγ and tumor necrosis factor alpha), production of IL-2 necessary for CD8^+^ T cell maintenance, and by providing help to B cells through CD40L co-stimulation (51). Although some tumor infiltrating B cells can be polarized towards an immunoregulatory phenotype, others retain inflammatory potential and contribute to cancer killing by acting as antigen presenting cells, section of tumor reactive antibodies, and production of cytotoxic factors (IFNγ and Fas Ligand) (52–54). The formation of organized aggregates of immune cells, called tertiary lymphoid structures (TLS), within the TME is increasingly recognized as important for productive B cell function and overall positive prognosis (55–57). TLS bring T cells, B cells, antigen presenting cells, and supportive stroma together to facilitate interactions necessary for optimized adaptive immune responses (57). Mature TLS contain germinal center reactions which are necessary for generating high-affinity B lineage plasma cells and memory cells (57). Recently, it was observed that TLS in renal cell carcinoma tumors generate cancer targeting plasma cells that disseminate across the TME *via* fibroblastic tracks (58). Therefore, TME immune activity has profound implications for overall disease outcome. Improved definition of local tissue immune dynamics and resolution of the origin and interaction of regulatory and inflammatory pathways will be essential for next generation cancer therapies.

### 2.2 Spatial organization in tumor immunity

A critical hallmark of tumor biology is the complexity and heterogeneity of cells within the 3D TME. Immense effort has been made to spatially analyze tumor biopsies, in addition to the more routine -omic analysis (genomic, proteomic, transcriptomic, metabolomic), to identify the role of each cell on cancer evolution (59–69). Significant progress has been made towards understanding how the spatial arrangement of immune cells within the TME can serve as prognostic indicators (59–61). For example, recent studies have identified a correlation between high CD3^+^ and CD8^+^ T cells and positive prognosis in early breast cancer patients (59, 60). It has also been demonstrated that the spatial distribution of these immune cells in the tumor can predict prognosis better than conventionally used tumor staging (59). Similarly, formation of TLS and subsequent dissemination of newly differentiated plasma cells is spatially constrained and dependent on fibroblast organization within the 3D TME (58). Therefore, experimental systems that incorporate spatial variables will be most useful for developing a comprehensive model of tumor immunity.

### 2.3 Dissecting adaptive immunity in 3D models

A hallmark of adaptive immunity is the vast potential for antigen recognition. Each lymphocyte expresses a unique T cell receptor (TCR) or B cell receptor (BCR) with specificity for a given antigen. Upon initial recognition of their cognate antigen, lymphocytes are able, in concert with additional signaling pathways, to proliferate and differentiate into either effector or memory cells. A key challenge for any *in vitro* model system is to model the dynamics of antigen specific activity. Incorporation of lymphocytes into 3D models can be done with autologous cells, however populations isolated from peripheral blood do not fully capture the immune repertoire established in the TME. Nonetheless, these platforms facilitate dissection of antigen independent functionality, especially cell trafficking, spatial dynamics, and interactions with stromal cells. Engineered systems where immune receptors with known antigen specificity are introduced into defined lymphocyte populations is another strategy that has already been widely implemented in 2D cultures (47). This approach may be particularly suited for preclinical investigation, for example to model behavior of chimeric antigen receptor (CAR) T cells (47, 70, 71).

## 3 3D bioprinting

A major challenge of spatially analyzing patient resected tumors is the immense heterogeneity across patients, making it difficult to get reproducible data (63). 3D bioprinting can facilitate our understanding of how the spatial arrangement of cells within the TME correlates to cancer evolution by reproducibly fabricating engineered tumors and observing disease progression in real time. Advancements in multiplex imaging of tissues has enabled the identification and spatial location of each cell within the tumor microenvironment and can serve as the roadmap for fabricating tumor replicas. While bioprinting currently enables the positioning of cells and biological material with microscale precision, technological advancements have moved bioprinting closer to replicating whole tissues at the cellular level.

**Figure 2:**
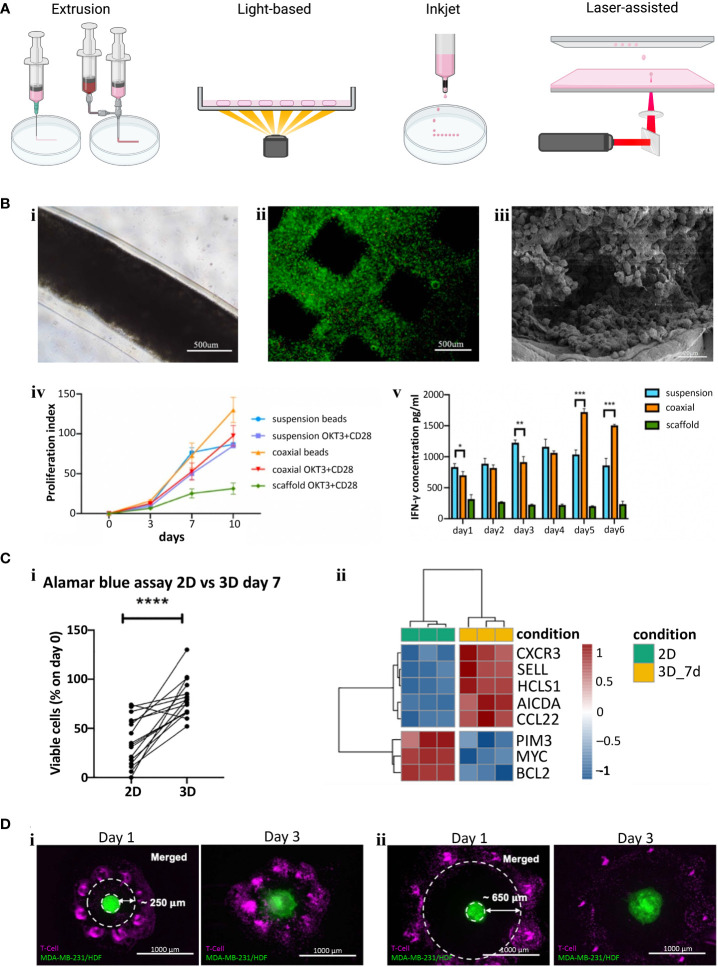
**(A)** Examples of different bioprinting methods. **(B)** Extrusion bioprinted T cells in gelatin-alginate hydrogel. i) Brightfield image of alginate + gelatin coaxial print on day 10. ii) Live/Dead viability stain (Calcein-AM/Propidium iodide) two hours post print. iii) SEM image of coaxial printed fiber showing T cells in the core 10 days after printing. iv) Proliferation index of T cells (Cells labeled with carboxyfluorescein succinimidyl ester (CFSE) were collected and analyzed *via* flow cytometry and proliferation index was calculated using ModFit software). v). IFN-γ secretion from CD4^+^ T cells (****p < 0.0001). Panel **(B)** adapted with permission from *Jin et al. (20*21*)*. Biofabrication, Copyright 2021, IOP Publishing Ltd (72). **(C)** Extrusion bioprinted MEC1 or primary CD19^+^ B cells. i) Percentages of viable leukemic primary cells, normalized to day zero, of 3D bioprints compared to traditional 2D cell culture (*p < 0.05, **p < 0.01, ***p < 0.001, ****p < 0.0001). ii) Heatmap summarizing genes of interest for chronic lymphocyte leukemia pathophysiology. Panel **(C)** adapted from *Sbrana et al. (20*21*)* (70). **(D)** A 3D bioprinted immune–cancer model containing a central MDA-MB-231/human dermal fibroblast spheroid with i) proximal, ~250 µm or ii) distal, ~650 µm T cell ring. Panel **(D)** adapted with permission from Dey et al. (2022). Biofabrication Copyright 2022, IOP Publishing Ltd (73).

There are four main types of bioprinting: extrusion, light, inkjet/microfluidic, and laser-assisted (**Figure 2A**). Extrusion bioprinting is the most common method of bioprinting which deposits materials or cells encapsulated in hydrogels onto a receiving platform. The resolution is dependent on the nozzle diameter and the viscosity of the material, generally ranging from 0.2 – 2 mm. A major advantage of extrusion bioprinting is the ability to rapidly switch between materials and cell types. Extrusion printing has been extensively used to generate bioengineered tumor models (74), but few studies have included T or B cells in the print. *Jin et al.* extrusion printed T cells encapsulated in an alginate + gelatin hydrogel to simulate a lymph node (72) (**Figure 2B**). The group also utilized coaxial printing to encapsulate T cells in an alginate hydrogel to create hollow channels that simulate lymphatic vessels. They found that the extrusion printed scaffold kept T cells in a resting state, while coaxial printing promoted T cell proliferation, stimulated CD8^+^ T cells, and slowed down T cell exhaustion (**Figure 2B**). Similarly, *Sbrana et al.* developed an extrusion printed chronic lymphocytic leukemia (CLL) model that maintained cell phenotype for 28 days, overcoming prior limitations of short cell survival and limited cell-cell interactions of traditional 2D approaches (**Figure 2C** **i**) (70). MEC1 (CLL cell line) or primary CD19^+^ B cells were encapsulated in CELLINK Laminink411, a proprietary hydrogel containing cellulose nanofibrils and alginate. RNAseq analysis supported their hypothesis that the bioprinted model better mimicked the physiological environment (**Figure 2C**). To create a tumor-immune interaction model, *Dey et al.* printed a ring of T cells into a collagen bath and deposited a single MDA-MB-231/human dermal fibroblast spheroid in the center (**Figure 2D**) (73). In each of the print designs, cancer cells migrated from the central spheroid and invaded into the collagen matrix. Prints with a proximal (250 µm) T cell ring had 70% less invasion compared to the control which contained no T cells, while prints with a distal (650 µm) T cell ring had 50% less invasion than control. They also found significantly higher expression levels of CCL2, IFNγ, and granzymes in proximal rings compared to distal rings. While the potential of bioprinting T and B cells has only just begun being explored, these studies provide evidence of improved physiological function within the 3D environment, meriting further investigation. Future studies may consider using bulk printing (extrusion or light based) combined with single cell printing (laser or inkjet/microfluidic) to systematically probe questions surrounding the spatial arrangement of T and B cells within the TME on cancer evolution.

Although 3D bioprinting of tumor-immune specific interactions has remained scarce, it is well acknowledged that 3D bioprinting enables the deposition of cells and biomaterials in spatially-defined patterns to create tissue-like architectures (75). Bioprinting can be utilized as a fabrication method for recapitulating the TME, taking advantage of multiple printing modalities as well as the ability to fabricate a 3D model using both scaffolds and scaffold-free methodologies (76). There are multiple advantages to bioprinting such as spatial control of the matrix properties (77), spatial distribution of biochemical factors (78), the ability to integrate perfusable vasculature (79), and high-throughput deposition of cells (80).

There are several examples of bioprinting tumor organoids to create model systems, including a recent study where *Chen et al.* bioprinted patient derived colorectal cancer organoids and healthy organoids to create microtissues for predicting drug therapy response (81). As another example, *Maloney et al.* used 3D bioprinting to develop a high-throughput drug screening method by immersion bioprinting patient-derived glioblastoma organoids into wells (82). However, neither of these examples explicitly study the role of immune cells. While 3D bioprinting has multiple advantages for precise tumor model development, the potential to bioprint tumor organoids with integrated adaptive immune elements is still an emerging area. *Gong et al.* demonstrated the use of an acoustic bioprinting technique to form mouse-derived bladder tumor organoids with an integrated T cell-containing immune microenvironment and additionally cocultured these organoids with autologous T cells to study immune cell interactions (83). Additional studies have included bioprinted non-organoid tumor cells with added adaptive immune components, such as the model developed by *Grunewald et al.* which details a 3D bioprinting method to create a neuroblastoma model with the addition of chimeric antigen receptor (CAR) T cells for immune interaction studies (71). The relative scarcity of bioprinted organoid systems that include adaptive immune components illustrates that this is a potential direction for future innovation. Bioprinted models are capable of including both patient-derived organoids and immune components (both endogenous and exogenous), and have potential to generate new model systems that recapitulate relevant cancer-adaptive immune interactions in spatially-defined microarchitectures.

## 4 Engineered organoids for modeling cancer-adaptive immune interactions

### 4.1 Organoids for modeling cancer

3D organoid cultures are highly relevant culture systems for modeling cancer as they can better recapitulate features of the native tumor microenvironment than 2D culture, including cell-cell interactions, extracellular matrix (ECM) interaction, and tissue composition and architecture. An organoid is defined as a 3D structure grown from stem cells to contain organ-specific cell types that has self-organized through cell sorting and spatially-controlled lineage commitment (84). Typically, cancer organoids are cultured from primary tumors where patient biopsies or surgical resections are digested into their components and cultured in a 3D matrix where they self-organize into organoids that reflect some important structural and functional properties of their parent organ. Organoids can also be grown using wild-type stem cells, which can also be useful for cancer modeling (85). Patient-derived tumor organoids (PDTOs) allow for functional testing, such as drug sensitivity, and can be used to correlate this data with an individual tumor’s genetic make-up (84). This organoid technology provides valuable models for studying cancer within an *in-vitro* system. PDTOs offer an *in-vitro* alternative to patient-derived tumor xenografts (PDTXs) where patient tumor tissue is implanted into an animal model and used to study patient-specific cancer progression and test therapeutic efficacy (85, 86).

**Figure 3:**
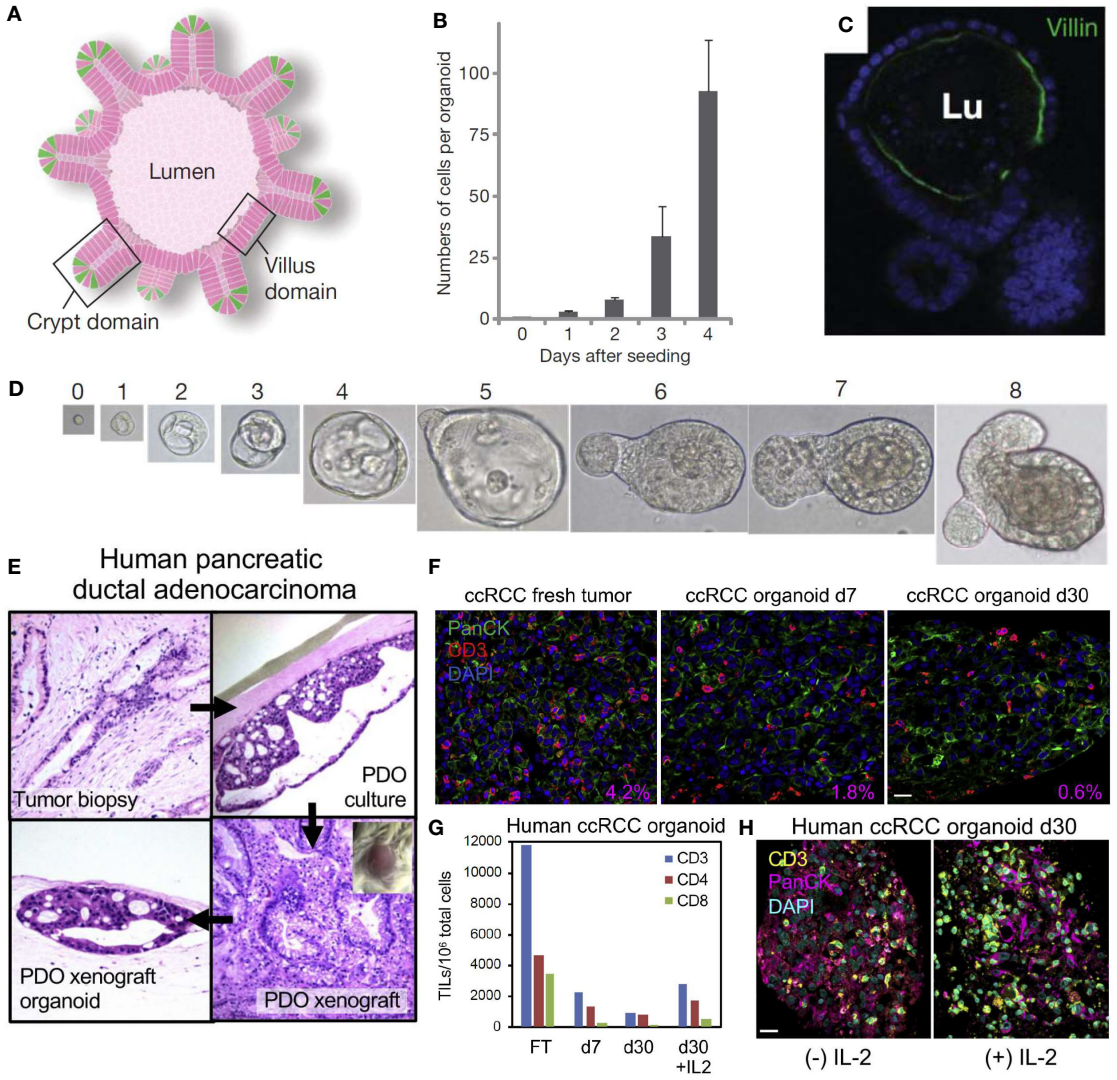
Self-organizing organoids derived from single stem cells, and the cellular composition of patient-derived organoid models of the tumor immune microenvironment. **(A)** Intestinal crypt organoid schematic showing villus-like epithelium lining the interior lumen (Lu), with crypt domains. **(B)** Average number of cells within crypt stem-cell derived organoids at 0-4 days after initial single-cell seeding. **(C)** Intestinal organoid confocal image showing villin in green (enterocytes) and nuclei in blue indicating that the organoid has formed a lumen and villus domain. **(D)** Growth from a single Lgr5-GFP^hi^ crypt stem cell at Day 0-8. Panels **(A–D)** reproduced from *Sato, et al. (* 87), Copyright 2009, with permission from Springer Nature Customer Service Center. **(E)** Demonstration of human pancreatic ductal adenocarcinoma (PDAC) patient-derived tumor organoid (PDTO, top right) cultured and xenografted to form a patient-derived tumor xenograft (bottom right) and re-derived as an organoid (bottom left). The original tumor’s histology (top left) has been recapitulated in the PDTO and the histology of the xenograft is preserved in the derived organoids. **(F)** Human clear cell renal cell carcinoma (ccRCC) PDTO in fresh tumor (Day 0), Day 7, and Day 30 with CD3^+^ tumor infiltrating lymphocytes (TIL) in red, PanCK in green, and nuclei in blue. The percentage area ratio of CD3^+^ cells in the lower right corner illustrates CD3^+^ cell content in fresh tumors and PDTOs. **(G)** Number of CD3, CD4, and CD8 TIL per 10^6^ organoid cells from fresh tumor (FT) and representative ccRCC PDTO at Day 7, Day 30, and Day 30 + interleukin-2 (IL-2). **(H)** Day 30 ccRCC PDTO with (+) IL-2 (100 IU/mL) and without (-) IL-2 stained for CD3 in yellow, PanCK in magenta, and nuclei in blue. All scale bars are 20 μm. Panels **(E–H)** reprinted from *Neal, et al.* (88), Copyright 2018, with permission from Elsevier.

In 2009, *Sato et al.* first demonstrated that 3D epithelial organoids can be derived from small biopsies in humans, while also demonstrating that these specimens can be expanded without genetic harm or limitations, as shown in **Figures 3A–D** (89). This research demonstrated that these PDTOs can more accurately represent the heterogeneous tumor environment, and showed a strikingly clear difference from mouse models, further illustrating the need for a representative human tumor model (85, 87, 89). In addition, PDTOs using adult stem cells have been used to establish organoids for a myriad of cancer types including but not limited to lung (90), colon (89), liver (91), pancreas (92, 93), prostate (94, 95), kidney (96), and breast (97) cancers.

### 4.2 Including immune elements in organoid models

PDTOs can recapitulate the tumor immune microenvironment by maintaining tissue architecture, stromal features, and the tumor’s endogenous immune cells, and through the addition of exogenous immune cells (87, 98–104). Solid tumors are composed of multiple stromal components such as ECM proteins, stromal cells including mesenchymal and immune cells, peptide factors, and the metabolites that stromal and cancer cells produce. In fact, cancer cells themselves may make up only 30% of the cells within the tumor (105, 106). There are two main methodologies for co-culturing immune cells and cancer organoids: adding exogenous immune cells to the organoids, or maintaining and expanding endogenous immune cells within the organoids. *Jenkins et al.* details a method of culturing organotypic tumor spheroids in a microfluidic device, where they were able to preserve both tumor cells and the endogenous immune cells (107). This method was able to retain both endogenous lymphocyte and myeloid populations and co-culture the tumor organoids with their native immune populations. Introducing exogenous immune elements to cancer organoid systems is another approach that can assist in making the models more complex and representative of *in vivo* conditions. As an example of this, *Tsai et al.* co-cultured pancreatic tumor organoids and patient-matched cancer-associated fibroblasts (CAFs) along with peripheral blood lymphocytes (108). Their T cells remained viable after 6 days in culture, and tumor-dependent activation of pancreatic stellate cells, which are myofibroblast-like CAFs that are prevalent in the TME, were also observed. *Dijkstra et al.* cultured colorectal and non-small cell lung carcinoma cancer PDTOs in a basement membrane with peripheral blood mononuclear cells (PBMCs) (109). This co-culture method enables enrichment of CD8^+^ tumor reactive populations from the patient peripheral blood. An alternative approach is to culture normal organoids with their endogenous stromal population and expose them to cancer cells. In a study done by *Zumwalde et al.* human epithelial breast tissue was used to generate ductal epithelial organoids with native stromal cells and resulted in 90% of the immune cell population being CD3^+^ T lymphocytes (110).

There are several different methods to culture tumor organoids, with and without immune cells. To culture these organoids, the cells are generally dissociated from the tumor, embedded in a matrix, and grown in the presence of growth media. Several common methods are used including Matrigel (murine tumor basement membrane extract) culture, air-liquid interface (ALI), microfluidic culture systems, and 3D bioprinting. With each organoid type, there are various stem-cell niches required to accurately recapitulate the organ, and these growth conditions are often facilitated through the addition of growth factor cocktails to mimic these niches (84).

The air-liquid interface (ALI) culture system embeds tumor organoids in a collagen gel where one surface contacts liquid culture and the other surface is exposed to air. The ALI method has shown endogenous immune cell populations surviving for more than 10 days in human lung and colorectal cancer (111). The ALI method was also used to culture patient-derived organoids from surgically resected tumors with the culture retaining the tumor epithelium, fibroblasts, and immune cells such as T helper cells, B cells, natural killer (NK) cells, and NK T cells for 30 days. Additionally, the heterogeneity of the T-cell receptors in the original tumors was recapitulated in this model (88). In **Figure 3E**, *Neal et al.* uses the ALI method to culture a human pancreatic ductal adenocarcinoma (PDAC) fresh tumor biopsy into a PDTO, and then xenografts the PTDO onto a mouse model to form a PDTX (88). These organoids retain the original histology from the fresh tumor and can also be re-derived as a PDTO after being xenografted. In addition, a fresh tumor of human clear cell renal cell carcinoma (ccRCC) can be cultured into a PDTO as shown in **Figures 3F, G**. These images stain for CD3^+^ tumor infiltrating lymphocyte (TIL) populations and detail the change in immune cell populations from fresh tumors and PDTOs at different time points. Addition of IL-2 also preserves the CD3^+^ cell population in ccRCC PDTOs, as shown in **Figure 3H** (88).

## 5 Organs on a chip for solid tumors-adaptive immune system interaction

Despite several advances in 3D tissue engineered *in vitro* models to mimic the TME, the complexity of the adaptive immune system and its role in the TME is still a challenge for biofabrication (75). First, the immune system is an interconnected network that includes different primary and secondary organs. Second, the immune system is directly linked to other key biological components, such as the lymphatic and vascular systems. Furthermore, several immune interactions associated with tumor antigen presentation, activation, response, and memory are dynamic, happening in different parts of the immune system, and continuously (112). To achieve these requirements, organs on-a-chip are exciting models that can replicate some of the multitypic responses of different organ systems *ex vivo* through microfluidic control to emulate the physiological and dynamic interactions between cells and tissues (113).

Microfluidic or “organ-on-a-chip” culture systems allow the possibility of precise control of microscale parameters that are poorly recapitulated using other technologies. These microfluidic technologies typically consist of transparent 3D microchannels that contain 3D microarchitecture of multiple tissue types and allow for stimulation by dynamic biomechanical forces (30). This method allows for the control of flow conditions, shear stresses, nutrient supply, and other parameters with control that is not afforded by other methods. Microfluidics can also allow for longer cultures of tumor organoids, as traditional cultures have limited nutrient supply and can result in the development of a necrotic core (114). Vasculature can be promoted in microfluidic devices to support long-term cultures (115).

These models are usually fabricated using elastomeric biomaterials, such as polydimethylsiloxane (PDMS) designed to connect channels and reservoirs, which allows extracellular matrices and biomimetics in 3D (116, 117). Over time, many organs on a chip have been developed to reproduce primary and secondary lymphoid organs, such as the thymus, the bone marrow, and the lymph nodes (118–120). Also, there are some reports of interaction between cancer cells and innate immune cells such as macrophages, T cells, and natural killers on a chip (121). Therefore, these advances open new possibilities to reconstruct the interactions of the adaptive immune system and cancer cells, improving drug screening and precision oncology.

In microfluidic culture, small sections of tumor tissue consisting of endogenous immune cells and tumor cells can be cultured intact, allowing for recapitulation of the patient tumor cell population and complexity (122). *Jenkins et al.* used this method to culture murine-derived and patient-derived organotypic tumor spheroids to study the immune response to immune checkpoint blockade (107). Another unique capability of microfluidic culture is the “body-on-a-chip” principle, where multiple organ-on-a-chip microfluidics can be connected to imitate the interactions between different organs. Using this principle, “metastasis-on-a-chip” methods have been used to mimic tumor spreading under controlled conditions. As an example of this system, *Aleman et al.* demonstrates the use of multiple microfluidic chambers hosting lung, liver, and endothelial cell populations (123). Colorectal cancer organoids in an interconnected main chamber are able to enter circulation and reach other organ sites to mimic and probe mechanisms of metastasis. While this work does not include immune cells, plans to include stroma and immune cells in organoid cultures have been reported. This method also allows for the probing of upstream and downstream effects of each culture chamber and the organ they represent, and inclusion of immune cells is also possible with this method.

### 5.1 Microfluidic models to study lymphocyte infiltration and migration

One of the challenges to mimic the TME *in vitro* is building systems that allow T cell infiltration (124). For instance, the desmoplastic profile of pancreatic cancer makes a challenge for T cell infiltration in the TME (125). To better understand this process, *Molica et al.* developed a model with a three-channel microfluidic device to investigate the T cell infiltration in a model of pancreatic cancer on-a-chip. The model was composed of a central channel filled with collagen and pancreatic adenocarcinoma cells and human umbilical vein endothelial cells (HUVEC) to emulate the vasculature on the other side (126). They isolated and seeded PBMC-derived human T cells on the endothelial side and found that the presence of the endothelial cells could be a barrier to prevent T cell infiltration in the main channel up to two days of incubation. They also observed that the T cell activation might contribute significantly to cell migration toward the central channel, especially in the presence of cancer cells.

In another study, researchers developed an organ on-a-chip system to understand the interaction between breast cancer cells (MDA-MB-231 and MCF7), T cells (TALL-104), and monocytes (THP-1) with hypoxia (127). The authors fabricated the chip by photopatterning and developed MCF7 and MDA-MB-231 spheroids in GelMA to create a 3D environment in the chip core. Monocytes and HUVEC cells were cultured surrounding the tumor spheroid to create a TME with an endothelial barrier. Using GelMA as a matrix, they designed an environment with controllable stiffness compatible with breast cancer matrix characteristics. It was observed that this engineered TME could attract T cells that were seeded surrounding the epithelial barrier. Also, T cell extravasation did not happen in the groups without endothelial barrier, and the groups without monocytes had a lower T cell infiltration. This data showed that the presence of different components of the TME is essential to understanding breast cancer biology *in vitro*. They also analyzed the potential for THP-1 polarization into macrophages M1, or M2, in the system. However, cancer cells did not significantly contribute to THP-1 cellular polarization, but upregulated some pro-inflammatory chemokines such as CCL4, CCL5, CCL11, and CXCL-8. Although some other cells such as stromal cells, T regulatory, and B cells were not present, this system opens new possibilities to interact with several cells involved with the TME.

### 5.2 Organs-on-a-chip to study lymphocytes cytotoxicity

**Figure 4:**
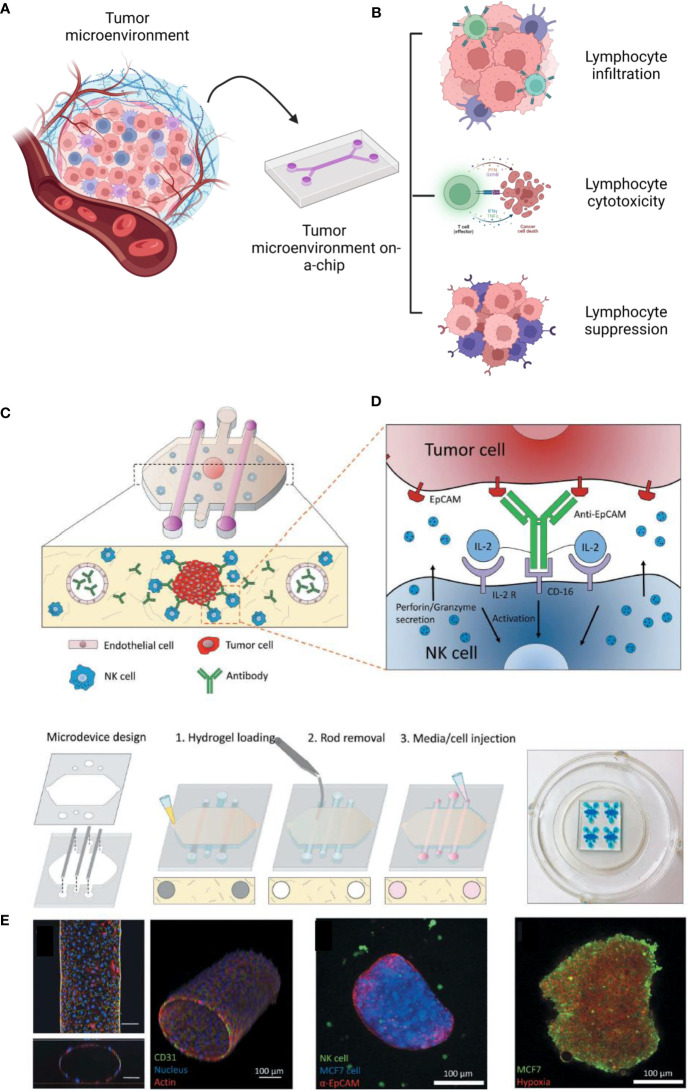
Diverse possibilities to study the tumor immune microenvironment on a chip **(A)**. These technologies have been reported to investigate lymphocyte infiltration, cytotoxicity and suppression **(B)**. As an example, reported by *Ayuso et al., 20*18, many cells, such as endothelial cells and natural killers, can be interacted on a chip **(C, D)**. The authors confirmed the formation of the endothelial barrier (by CD31), the cancer spheroid (coated with antibodies EpCAM) **(E)**. The spheroids were cultivated under hypoxia (with a sensing dye in red). This figure was partially created by Biorender.com and adapted with permission from *Ayuso et al., 2018* (130) (Taylor & Francis Group).

One of the most critical responses of lymphocytes related to the TME is cytotoxicity, mainly regulated by T CD8^+^ and natural killers (128). Natural killers are known for expressing CD16, which recognizes tumor cells and causes antibody-dependence cell cytotoxicity (129). To replicate these interactions *in vitro*, (**Figure 4**) *Ayuso et al.* developed a model to evaluate the natural killer cytotoxicity against breast cancer spheroids in a 3D matrix. The authors designed a microfluidic model containing breast cancer spheroids (MCF7 cells) in a collagen hydrogel and two lateral lumens coated with endothelial cells (HUVECs). It was observed that antibodies could be diffused by the biofabricated lumen more slowly than without the presence of endothelial cells, showing that the endothelial barrier was successfully formed. Although NK cells could access the spheroids before the antibodies, the association of antibodies and IL-2 was responsible for enhancing the NK-cytotoxicity after 24 hours. By time lapse imaging, it is possible to see NK traveling through the 3D collagen matrix toward the tumor spheroids (130). Most recently, *Roteix et al.* developed a microfluidic device to understand the dynamic interactions of T cells and melanoma spheroids. Their device was composed of a droplet-generating region followed by a trapping region to cultivate, maintain, and monitor cells over time. With this technology, it was possible to biofabricate small spheroids (35-45 microns) in 234 anchors, allowing CD8^+^ T cells to travel through the chip in a controllable system. By mathematical modeling, the authors found that the T cell cytotoxicity is dependent on cellular cooperation and that the short- and long-term interactions between cytotoxic T lymphocytes are key factors in determining an effective immune response against tumor spheroids (131).

### 5.3 Organs-on-a-chip to study cancer resistance and immune suppression

Monocytes are responsible for lymphocyte chemoattraction in many types of cancer (132). Nevertheless, in the hepatitis B virus-related hepatocellular carcinoma tumor microenvironment, monocytes inhibit T cell functions *via* PDL-1 signaling (133). To better understand this process on-a-chip, *Lee et al.* developed a model involving a central channel composed of human hepatocellular carcinoma cells and monocytes immersed in a collagen 3D matrix and lateral channels containing T cells. It was observed that the presence of monocytes primarily associated with hepatocellular carcinoma cell aggregates was responsible for increasing the expression of PD-1 in T cells. It was also observed that the suppression of monocytes on T cell cytotoxicity toward cancer cells was significantly lower on the 2D model, showing that a 3D complex system represents better the actual pathological events (134).

## 6 Challenges and opportunities of using 3D models to study the efficacy of cancer therapies

The use of three-dimensional (3D) models as an alternative to traditional 2D cell cultures and animal models has become a promising approach to studying cancer biology and evaluating novel therapies (135). Nevertheless, there are several challenges that must be overcome when using 3D models to assess the efficacy of immunotherapies, including oncolytic virus therapy, vaccines, and T-cell therapies (1).

One major challenge is the dense extracellular matrix (ECM) of 3D tumors, which is difficult to replicate *in vitro*, but is a key component to mimic, since it can restrict the penetration of therapeutic agents. The ECM is a complex network of proteins and other molecules that provides structural support to tissues and organs, and it can act as a barrier to the diffusion of therapeutic agents (136). For instance, the compact and stiff ECM in 3D models may impede the spread of oncolytic viruses that target and kill cancer cells. Additionally, the tumor microenvironment in current 3D models may not accurately reflect the complexity of human tumors, which can include diverse populations of immune and stromal cells. T-cells, for example, are critical in the effectiveness of several immunotherapies; however, the complexity of T-cell interactions with the tumor microenvironment in current 3D models makes it challenging to predict the efficacy of T-cell-based therapies (137). Despite these challenges, 3D models offer numerous advantages over traditional cell cultures and animal models, such as better replication of physiological conditions of tumors and high-throughput drug screening. Several studies have demonstrated the efficacy of 3D models in studying chemotherapeutic drugs like paclitaxel and doxorubicin in breast cancer and glioblastoma (25).

Recent studies have shown that 3D models of human tissues could aid in understanding immune-cancer interactions and evaluating the effectiveness of T-cell-based therapies. In one study, *Dey et al.* developed 3D tumor-T cell platforms that allowed for the study of complex immune-cancer interactions and the evaluation of genetically engineered CD8^+^ T cells expressing mucosal-associated invariant T (MAIT) cell receptors against breast cancer cells (73). Their study showed that the engineered T cells effectively eliminated tumors in 3D culture. Another study by *Wie et al.* used a recombinant vaccinia virus encoding an EpCAM BiTE to enhance antitumor immunity and modulate the immune suppressive microenvironment in several solid tumors, providing preclinical evidence for the therapeutic potential of VV-EpCAM BiTE (138).

Despite the challenges associated with using 3D models to evaluate immunotherapies, the development and optimization of these models are essential. Accurately replicating the complexity of the tumor microenvironment and the ability of immunotherapeutic agents to penetrate the ECM are critical factors to consider. Ultimately, successful development of 3D models that allow for the testing of immunotherapies could accelerate drug discovery and development while reducing animal testing and ethical concerns, providing a reliable platform for predicting clinical outcomes in humans.

## 7 Perspectives and conclusion

The intersections of biofabrication and tissue immunity are only beginning to emerge, but the possibilities for leveraging advanced tissue engineering methods in order to gain improved insights into the interactions between immune cells and healthy (or diseased) tissues are tangible. Here we reviewed a few strategies that have demonstrated the potential to control tissue architecture, composition, and function to more precisely dissect the complex regulation of the immune system in the presence of other cell types in the TME. While specific reports taking advantage of the entire breadth of biofabrication tools specifically to address cancer adaptive immunity remain relatively scarce (in comparison to innate immunity at least), it is easy to foresee how biofabrication can be relevant in this area.

**Figure 5:**
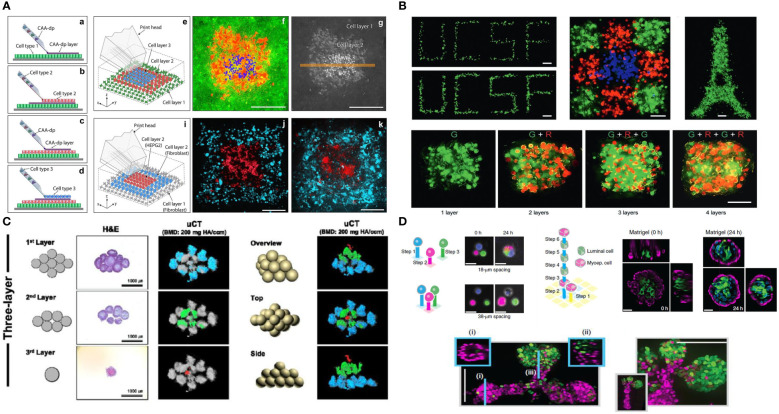
Future possibilities with single cell patterning of the tumor microenvironment **(A)**. Direct 3D cell bioprinting, open-volume microfluidics to position individual cells in a complex 3D patterns panel **(A)** adapted from *Jeffries, et al.*(2020) Scientific Reports (144) **(B)**. High-definition single-cell printing droplet method for cell by cell fabrication of biological structures panel **(B)** reproduced from *Zhang, et al.*(2020) Advanced Materials (145). **(C)**. 3D bioprinting of spheroids, 3D Bioprinting of hMSC/HUVEC spheroids *via* Aspiration-assisted Bioprinting panel **(C)** adapted with permission from *Heo et al.* (2020) Biofabrication Copyright 2020, IOP Publishing Ltd (146). **(D)** DNA-programmed assembly of cells (DPAC), reconstituting the multicellular organization of organoid-like tissues with programmed size, shape, composition and spatial heterogeneity panel **(D)** adapted with permission from *Todhunter, et al.*(2015) Nature Methods, Copyright 2015, Nature Publishing Group (147).

The field of 3D bioprinting, for instance, has evolved at a very high pace to address limitations of printing resolution, precision and throughput; all which play a major role in enabling biologists and engineers to address immune biology-specific questions. For instance, while previous works to understand cell-cell communication in the field have concentrated on the interactions of co-cultures of various cell types randomly distributed in extruded hydrogels or scaffold-free cell aggregates, expanding efforts have been dedicated towards mimicking tissue microenvironments with single cell precision, or in the context of cell neighborhood. The ability of replicating the exact location of adaptive immune cells relative to resident cells in TME, allows one to ask questions relative to spatial heterogeneity, paracrine and juxtacrine communication, cell activation and many other aspects that are key to regulating cancer immunity. Similarly, the ability of engineering neighborhoods of cells, allows for the fabrication of “hot” or “cold” tumors, which have been demonstrated to play a major effect on cancer progression and survival. Research has demonstrated, for instance, that often it is not only the presence of immune cells in the TME that determine tumor evolution, but rather the type of cells and their distribution in specific regions within a tumor, such as the tumor border or the edge (139). In fact, recent reports have shown that the exact location of B and T cells can be a better determinant of tumor staging than the traditional staging criterial currently used in the clinic (140–142). It is virtually impossible to replicate this level of precision relative to the spatial pattern of immune cell communication with the tumor using conventional culture models. Therefore, advanced biofabrication, especially methods that enable precision patterning of single immune cells, or small groups of cells and tumor cells, are likely to advance the field substantially in the near future. This remains a major focus of our group and others (143) (**Figure 5**).

Another aspect that emphasizes the significance of biofabrication to the field of adaptive immune biology, is the ability of controlling longitudinal physical variables, such as fluid flow, mechanical loading, and other parameters, using microfluidics. This, when combined with organoid fabrication or bioprinting, offers a unique longitudinal view of biological systems that is difficult to access in either two-dimensional culture models or in animals. Organs on-a-chip have been praised for their ability to maintain tissue constructs in near physiologic conditions for prolonged periods of time, under a microscope, while biological events unfold under direct observation of cell behavior in real-time. This offers an additional dimension of time to previously observed immune biology mechanisms that could not be observed longitudinally. Examples of such biological systems in simple models have been reported many times in the organ-on-a-chip literature. Moreover, these can be observed in the context in which they occur in the body, such as relative to the migration of cells through blood capillaries, crossing the basement membrane, invading into a tumor mass, and etc. Therefore, the possibilities with integrated organ on-a-chip models with other tissue fabrication approaches is warranted for improved approaches in adaptive immunity and cancer research.

In short, it is evident that the complexity of the interactions between adaptive immunity and cancer are too complex to elucidate using simple and static 2D models. The mechanisms regulating tumor initiation, progression and response to treatments are just too site-specific and too dynamic in 3D. The difficulty of replicating human immunity in animal models also makes advanced biofabrication model systems particularly exciting for cancer biology and immunity research, given the ability of manipulating primary human cells for virtually any desirable aspect of cancer biofabrication. The requirements towards expanding and implementing these methods in day-to-day cancer biology research are only beginning to emerge, and certainly those are related to the ability of truly mimicking tumor/immune complexity *in vitro*. The examples proposed here provide some evidence of the capabilities of these engineered models.

Likely the ability of controlling tissue and cellular spatial distributions with precision, of culturing multi-typic tissue cocultures over prolonged periods of time, and under conditions that better represent the human body, will further attract the attention of immune and cancer biologists. Likewise, the evolution of engineered models to further integrate various organ systems, such as the existing efforts invested towards the body on-a-chip, or to combine engineered tissues with biosensors, real-time quantitative read-outs, and other methods of genetic manipulation of tissue function, will offer a major leap in performance of these engineered models relative to their static 2D *in vitro* counterparts. This will certainly attract even greater attention and provide further impetus for the adoption of biofabricated models for cancer and adaptive immunity research in the near future. In the meantime, it is important to also highlight that these engineered models are only as good as their validation against well-established and understood model systems, and actual patient response. Therefore, significant effort should be made towards addressing these milestones. Recent efforts, especially in the areas of organs on-a-chip and organoid models, have been offered major steps towards demonstrating the efficacy of these models in matching clinical trial data in patients (148), and more of that is encouraged in the field. This will pave the way for future research on biofabricated models of adaptive immunity and cancer.

## Author contributions

RV wrote the introduction, ML and CS wrote the organoid section, MS wrote the organs on a chip section, HH wrote the bioprinting section, JM, and AS wrote the adaptive immune section and LB wrote the perspective and conclusion. All authors contributed to the article and approved the submitted version.
